# Integration of biomimetic organoid-on-chip and 2D models advances the mechanistic Understanding of STEAP3-mediated regulation in intestinal viral infection

**DOI:** 10.1038/s41598-025-15846-4

**Published:** 2025-08-20

**Authors:** Yi-Wen Chen, Huan-Jung Chiang, Kuan-Ting Liu, Chun-Wei Kao, Shan-Ren Xie, Chao-Ming Su, Yu-Yin Shih

**Affiliations:** 1 Research and Development Center for x-Dimensional Extracellular Vesicles, China Medical University Hospital, Taichung, 404332 Taiwan; 2 Department of Bioinformatics and Medical Engineering, Asia University, Taichung, 41354 Taiwan; 3 Graduate Institute of Biomedical Sciences, China Medical University, Taichung, 406040 Taiwan; 4 Research Center for Emerging Viral Infections, College of Medicine, Chang Gung University, Taoyuan, 33302 Taiwan; 5 Department of Medical Biotechnology & Laboratory Science, College of Medicine, Chang Gung University, Taoyuan, 33302 Taiwan; 6 Department of Microbiology and Immunology, School of Medicine, Stanford University, Stanford, CA 94305 U.S.

**Keywords:** Organoid, STEAP3, Viral infection, SARS-CoV-2, Biomimetic, Chip, Translational research, Biomedical engineering

## Abstract

Traditional investigations of viral infection mechanisms have predominantly relied on two-dimensional (2D) cell culture models, which lack the structural organization and physiological relevance of native tissues. These systems often fail to capture key features such as spatial cell–cell interactions, tissue-specific heterogeneity, and microenvironmental complexity that govern virus–host dynamics *in vivo*. To address these limitations, we established an integrative platform that combines the strengths of both 2D and three-dimensional (3D) models to investigate the role of six-transmembrane epithelial antigen of prostate 3 (STEAP3), a membrane ferrireductase, in regulating viral infection in human intestinal epithelium. The 2D system enabled high-resolution mechanistic interrogation of STEAP3-dependent viral entry processes, while the patient-derived 3D colon organoid model recapitulated the architectural and cellular complexity of intestinal tissue, allowing spatially resolved assessment of infection patterns. Using this integrated approach, we found that *STEAP3* knockdown significantly increased viral entry and infection, particularly in enterocytes and enteroendocrine cells. To further mimic physiological conditions in human body, we developed a vascularized organoid-on-chip model, in which increased viral signals were observed within vascular lumens upon *STEAP3* depletion, suggesting a protective role of STEAP3 in limiting viral dissemination. For efficient and multiplexed screening of antiviral mechanisms, we also fabricated a 3D-printed 27-well chip tailored for organoid culture. By leveraging the complementary advantages of both 2D and 3D systems, this study demonstrates the power of integrated biomimetic modeling platforms to investigate antiviral defense mechanisms and underscores their value for engineering physiologically relevant infection models.

**Supplementary Information:**

The online version contains supplementary material available at 10.1038/s41598-025-15846-4.

## Introduction

Six-transmembrane epithelial antigen of prostate 3 (STEAP3) is a membrane-associated protein with six transmembrane domains at its C-terminus and an N-terminal oxidoreductase domain. It is localized in the plasma and endosomal membranes, where it plays a key role in intracellular iron metabolism^1^. Acting as an endosomal oxidoreductase, STEAP3 facilitates the reduction of ferric iron (Fe³⁺) to ferrous iron (Fe²⁺), which is then transported into the cytoplasm for cellular utilization^2^. Mice with a spontaneous STEAP3 deletion exhibit severe microcytic anemia, highlighting its essential role in iron homeostasis^3^. Recent studies have identified STEAP3 as a key regulator of ferroptosis, an iron-dependent form of cell death, with significant implications for tumorigenesis^4,5^. The presence of a p53 binding site in the promoter region of STEAP3 suggests that its expression is regulated by p53 activation, which promotes apoptotic signaling^6,7^. Beyond its roles in apoptosis and ferroptosis, STEAP3 functions as a negative regulator of cell cycle progression by interacting with the Nix and Myt1 proteins^8^. Therefore, in addition to its well-established role in regulating iron homeostasis, STEAP3 also contributes to various cellular functions, particularly those involved in cell death-related pathways.

Numerous viruses, including enteroviruses and coronaviruses, infect the gastrointestinal (GI) tract, where they replicate within the intestinal epithelium and disrupt ion homeostasis, leading to gastrointestinal symptoms such as diarrhea. Notably, SARS-CoV-2 emerged in late 2019, leading to the global coronavirus disease 2019 (COVID-19) pandemic and causing over 7 million deaths^9^. Some COVID-19 patients presented with GI symptoms such as diarrhea, vomiting, and abdominal pain^10^. while SARS-CoV-2 viral RNA remained detectable in fecal samples for a longer duration than in respiratory samples^11^. Given that the GI tract is composed of diverse cell types that coordinate to regulate physiological functions, including nutrient absorption and the secretion of water and enzymes^12^, to comprehensively investigate the underlying mechanisms of viral infection in GI tract could advance medical interventions.

However, conventional two-dimensional cell cultures lack the capability to replicate the 3D architecture of intestinal tissues and the heterogeneous expression of distinct cell types. Recently, 3D models have emerged as a promising system for investigating disease progression and the complex interactions between microorganisms and their hosts^13,14^. Organoid is a typical 3D culture system that recapitulate the genetic and phenotypic features of the organs within human body. They can grow and differentiate from embryonic or adult stem cells and self-organize into 3D structures in the appropriate cultured condition^15^. Because organoid systems faithfully retain the key features of native tissues, they serve as powerful models for precisely investigating host-pathogen interactions.

In this study, we leveraged both 3D and 2D model systems to comprehensively investigate the role of STEAP3 in regulating viral infection in intestinal tissues. Our findings demonstrate that STEAP3 exerts an inhibitory effect on viral infection, particularly during the viral entry phase, possibly through interactions with viral receptors. To facilitate high-throughput and reproducible experimentation, we employed a custom-designed 27-well colon organoids-on-chip platform, which allows simultaneous analysis of multiple conditions within a single batch. Using this system, we observed that *STEAP3* deficiency markedly enhanced viral infection in intestinal epithelium, especially enterocytes and enteroendocrine cells. To better recapitulate physiologically relevant conditions and account for the influence of the tissue microenvironment, we further developed a vascularized version of the chip. In this vascularized colon organoids-on-chip model, *STEAP3* knockdown led to increased viral infection within vascular lumens, suggesting that loss of STEAP3 may facilitate viral dissemination through the circulatory system. Collectively, these findings highlight the utility of colon organoids-on-chip as a biomimetic platform for studying STEAP3-mediated antiviral mechanisms in the intestinal epithelium. Our results suggest that *STEAP3* deficiency increases the susceptibility of both intestinal tissues and their surrounding microenvironment to viral infection. Thus, the integration of 2D and 3D models provides a robust framework for elucidating the antiviral functions of STEAP3 in the context of intestinal viral pathogenesis.

## Materials and methods

### Reagents and antibodies

Ferric Ammonium Citrate (FAC ;F5879) was purchased from Sigma. Antibodies against STEAP3 (sc-376327), SCARB2 (sc-55571), and His (sc-8036) were from Santa Cruz Biotechnology. Anti-EV-A71(MAB979), anti-Actin (MAB1501), anti-Flag (F3165) antibodies were from Sigma. ACE2 antibody (AF933) was from R&D systems. Anti-E-cadherin (ab40772) and anti-MUC-2 (ab11197) antibodies were from Abcam. LGR5 antibody (A12327) was from ABclonal. Anti- MUC-2 (GTX637372) and anti- LGR5 (GTX631935) antibodies were from GeneTex.

### Cell culture, virus, SiRNA and plasmids

Rhabdomyosarcoma (RD), Caco-2, human embryonic kidney 293 cells expressing SV40 large T antigen (HEK293T) cells, and calu-3 cells were purchased from the American Type Culture Collection (ATCC) and were grown in DMEM containing 10% fetal bovine serum (FBS). Enterovirus A71 (EV-A71; Tainan/4643/98) was used to infect RD cells in this study. The pseudotyped SARS-CoV-2 S viruses (SARS-CoV-2 S entry viruses), expressing the Spike protein in its wild-type (WT) form or with mutations (D614G, B.1.1.7, and V501Y.V2), along with a luciferase reporter, were obtained from the National RNAi Core Facility, Academia Sinica, Taiwan. In order to simultaneously detect viral infectivity by two different methods, we have designed the SARS-CoV-2 S entry virus expressing Spike protein and dual reporters of green fluorescent protein (GFP) and luciferase (GFP-SARS-CoV-2 S entry virus). This virus was generated by the National RNAi Core Facility, Academia Sinica, Taiwan. SARS-CoV-2/human/TWN/CGMH-CGU-01/2020 isolate was used to study the effect of STEAP3 on live virus infection. For siRNA transfection, cells or organoids were transfected with siRNA targeting to control (5’-GAUCAUACGUGCGAUCAGA-3’) or *STEAP3* (5’-GGGAGUUCAGCUUCGUUCA-3’) purchased from Sigma-Aldrich and Lipofectamine RNAiMAX reagent (Thermo Fisher Scientific). The protocol for transfection of organoids with siRNA was modified from the previous report^16^. To ectopically express *SCARB2* or *STEAP3* in HEK293T cells, cells were transfected with pcDNA3.1-His/Myc-*SCARB2* or pIRES2-Flag-*STEAP3* constructed from Blossom company, Taiwan and Lipofectamine 3000 transfection reagents.

### Plaque assay

RD cells grown in 6-well plate at 90% confluence were infected with virus at 10-fold serial dilutions for 1 h. After washed with phosphate-buffered saline (PBS), cells were maintained in 2% FBS/DMEM medium with 0.4% of agarose for 3 days. To visualize the plaques, infectious particles, cells were fixed with 10% formalin and stained with 0.5% crystal violet.

### Luciferase activity assay

Caco-2 cells and colon organoids were infected with the SARS-CoV-2 S entry virus carrying a luciferase reporter (1.1 × 10⁶ relative infection units) in the presence of polybrene (8 µg/ml) for 24 h. Total protein was extracted using Glo Lysis Buffer (Promega), and luminescence signals were measured using the ONE-Glo luciferase assay (Promega) on a Synergy 2 microplate reader (BioTek). The relative luciferase activity was quantified as the ratio of luminescence signal to protein concentration and analyzed.

### Virus binding and internalization assay

For binding assay, RD cells were grown in 6-well plate and transfected with siRNA for 48 h. Transfected cells were infected with EV-A71 (MOI = 1) at 4 °C for 1 h, and then washed with cold PBS for three times. The membrane-bound viruses were determined by real-time PCR. The internalization assay was conducted after virus attachment to cells at 4 °C for 1 h. Infected cells were incubated at 37 °C to another 1 h, and 0.05% trypsin was used to detach virus from cell surface. Intracellular viruses were measured by real-time PCR.

### Viral RNA extraction and real-time PCR (RT-PCR)

Total RNA was extracted using the TRIzol reagent (Thermo Fisher Scientific). RNA of live virus was isolated using the LabTurbo 48 Compact System (Taigen Bioscience) with LabTurbo Viral DNA/RNAMini kit. Purified RNAs (1 µg) were reverse transcribed to cDNA using ReverTra Ace (TOYOBO). The relative gene expression was determined by using LightCycler 480 Real-Time PCR System (Roche) and KAPA SYBR FAST qPCR master mix (Kapa Biosystems) with specific primers. The primers used for targeting EV-A71 (Tainan/4643/98) and live SARS-CoV-2/human/TWN/CGMH-CGU-01/2020 were described in the previous study^17^.

### Immunoprecipitation (IP) assay

Total protein was extracted from cells or colon cancer organoids using lysis buffer (20 mM Tris-HCl, 137 mM NaCl, 10% Glycerol, and 1% NP-40) and subjected to immunoprecipitation with indicated antibodies at 4 °C overnight. After wash with PBS for three times, the precipitates were dissolved in SDS-PAGE and Western blotting with specific antibodies.

### Immunofluorescence staining assay

Colon cancer organoids were fixed with 4% paraformaldehyde and subsequently permeabilized with 0.1% Triton X-100. Following three washes with PBS, the organoids were blocked with 2% BSA for 1 h. They were then stained with the specified antibodies and counterstained with 4,6-diamidino-2-phenylindole (DAPI). Fluorescence images were captured using a Dragonfly confocal microscope (Andor). The stained area was quantified using ImageJ software (National Institutes of Health) from at least five fields per group. Similarly, within these fields, the number of green fluorescent dots representing GFP-SARS-CoV-2 S entry virus was counted. To assess the susceptibility of different intestinal epithelial cell types to viral infection following STEAP3 knockdown, the ratio of GFP signals from SARS-CoV-2 S entry viruses to the positive staining area of individual intestinal markers was calculated. Results are expressed as the mean percentage (± s.d.), and statistical analysis was conducted using one-way ANOVA.

### Fabrication of a 27-well chip

A 27-well chip was fabricated using a stereolithographic 3D printer (MiiCRAFT) with MiiCraft BV007a Clear resin (CADworks3D), a polydimethylsiloxane (PDMS) alternative that exhibits reduced autofluorescence. Following fabrication, the printed chip was dried and subjected to UV post-curing for 5 min using the FormCure system (FormLabs). The cured chip was then mounted onto a 1 mm-thick cover glass and rinsed with distilled water.

### Establishment of colon cancer organoids from patient-derived tumor specimens

**Fig. 1:**
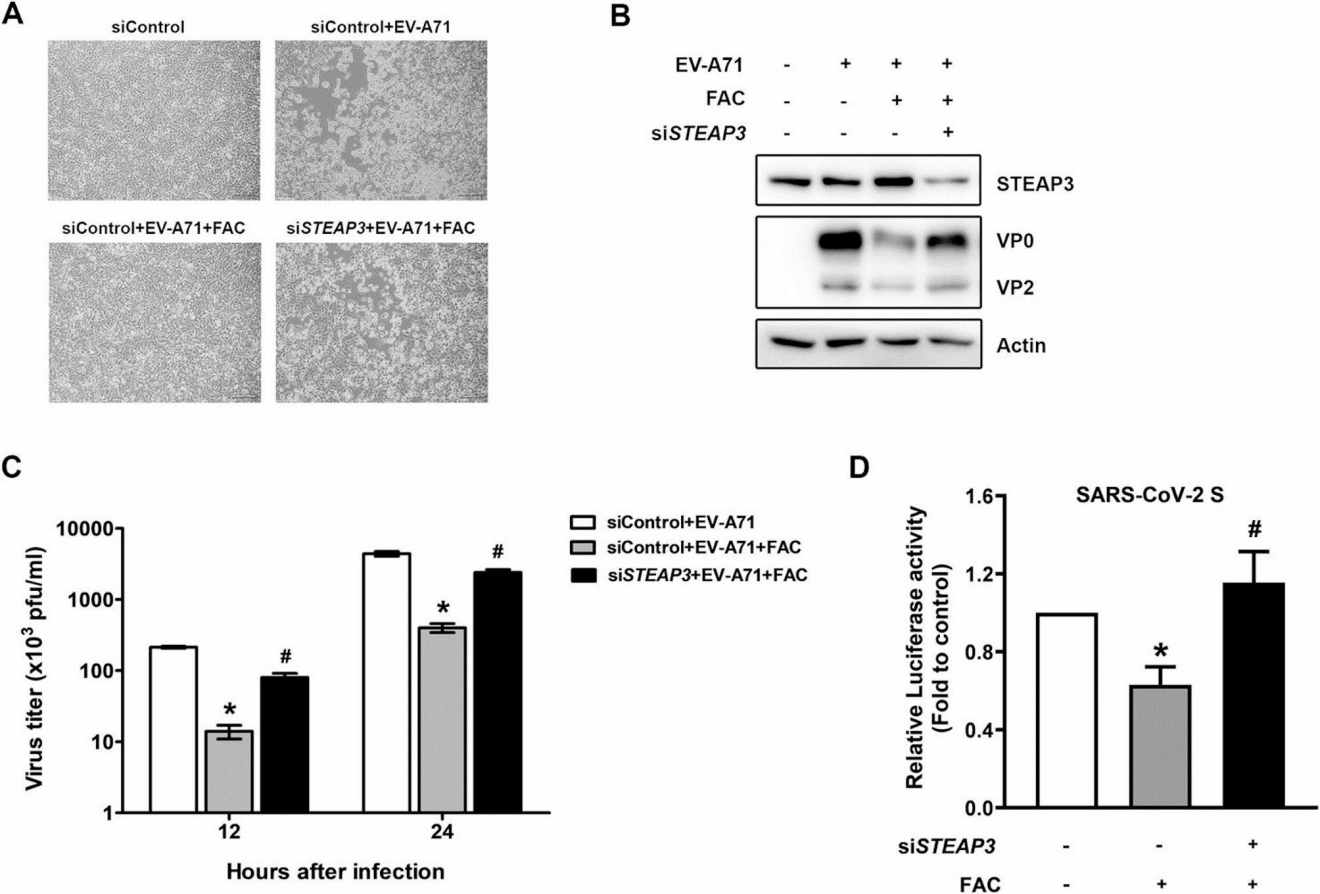
STEAP3 is involved in FAC-mediated antiviral effects. (**A**) siControl or si*STEAP3*-transfected RD cells were infected with EV-A71 (MOI = 10^−3^) with or without FAC (100 µM) for 24 h. The CPE was visualized using bright-field microscopy. Scale bar = 100 μm. (**B**) The total cell lysate was extracted from siRNA-transfected RD cells infected with EV-A71 (MOI = 10^−3^) for 24 h, resolved by SDS-PAGE, and then analyzed by immunoblotting with the indicated antibodies. The uncropped and unprocessed blots are provided in Supplementary Fig. S16. (**C**) The virus titer was measured from siRNA-transfected RD cells infected with EV-A71 (MOI = 10^−3^) in the presence or absence of FAC for 12 and 24 h. *p < 0.05 versus siControl-transfected cells without FAC treatment. ^#^*p* < 0.05 compared to siControl-transfected cells with FAC treatment using the student’s *t*-test. All data are shown as the mean (± s.d.). (**D**) Caco-2 cells expressing siRNA targeting to control or *STEAP3* were infected with SARS-CoV-2 S entry virus for 24 h with or without FAC treatment. The total cell lysate was collected, and the luciferase activity was measured.

This study was approved by the Research Ethics Committee of China Medical University Hospital (CMUH-REC1-083), Taiwan. All research procedures were conducted in accordance with approved protocols. All samples were obtained with written informed consent from participants, and all methods were carried out in accordance with relevant guidelines and regulations, including the Declaration of Helsinki. Colon cancer specimens were obtained from patients undergoing surgical resection at China Medical University Hospital, Taiwan. The resected specimens were cut into small fragments (approximately 2 mm in size) and enzymatically digested with 5 mg/ml collagenase at 37 °C for 2 h. The resulting cell suspension was washed multiple times with PBS and passed through a 40 μm cell strainer (Corning) to isolate single cells. The collected cells were then suspended in Geltrex (Thermo Fisher Scientific) and seeded onto a 27-well chip, where they were incubated at 37 °C for 30 min to allow gelation. The culture medium formulation for colon organoids consisted of advanced DMEM/F12 medium (Gibco) supplemented with 1X B-27 (Gibco), 1X N2 (Gibco), 50 ng/ml EGF (ProSpec), 500 ng/ml R-spondin-1 (ProSpec), 100 ng/ml Noggin (ProSpec), 10 µM SB202190 (Sigma), 500 nM A83-01 (Sigma), 1 mM N-acetylcysteine (Sigma), 10 µM Y-27,632, and 10 mM nicotinamide (Sigma). The colon organoid was formed after 10–14 days, the morphology and specific colon markers have been identified as shown in Fig. 1a.

### Construction of vascularized colon organoids in a chip

Colon cancer organoids embedded in a Geltrex matrix were cultured on the 27-well chip at 37 °C for 30 min to allow gelation. Following this, human umbilical vein endothelial cells (HUVECs) and human dermal fibroblasts (HDFs) were seeded onto the pre-established colon cancer organoids within the Geltrex dome, in the presence of 10 mg/ml fibrin and 0.5% adipose-derived extracellular matrix (AdECM), and incubated for an additional 30 min. A mixed medium composed of colon tumoroid growth medium and EGM™−2 Endothelial Cell Growth Medium in a 1:1 ratio was then added. After five days of co-culture, a vascular network was established.

### Statical analysis

Result was analyzed using GraphPad Prism 5 software (GraphPad Software). Results are expressed as the mean ± s.d. from three independent experiments.

## Results

### *STEAP3* deficiency impaired the antiviral effects of FAC

**Fig. 2:**
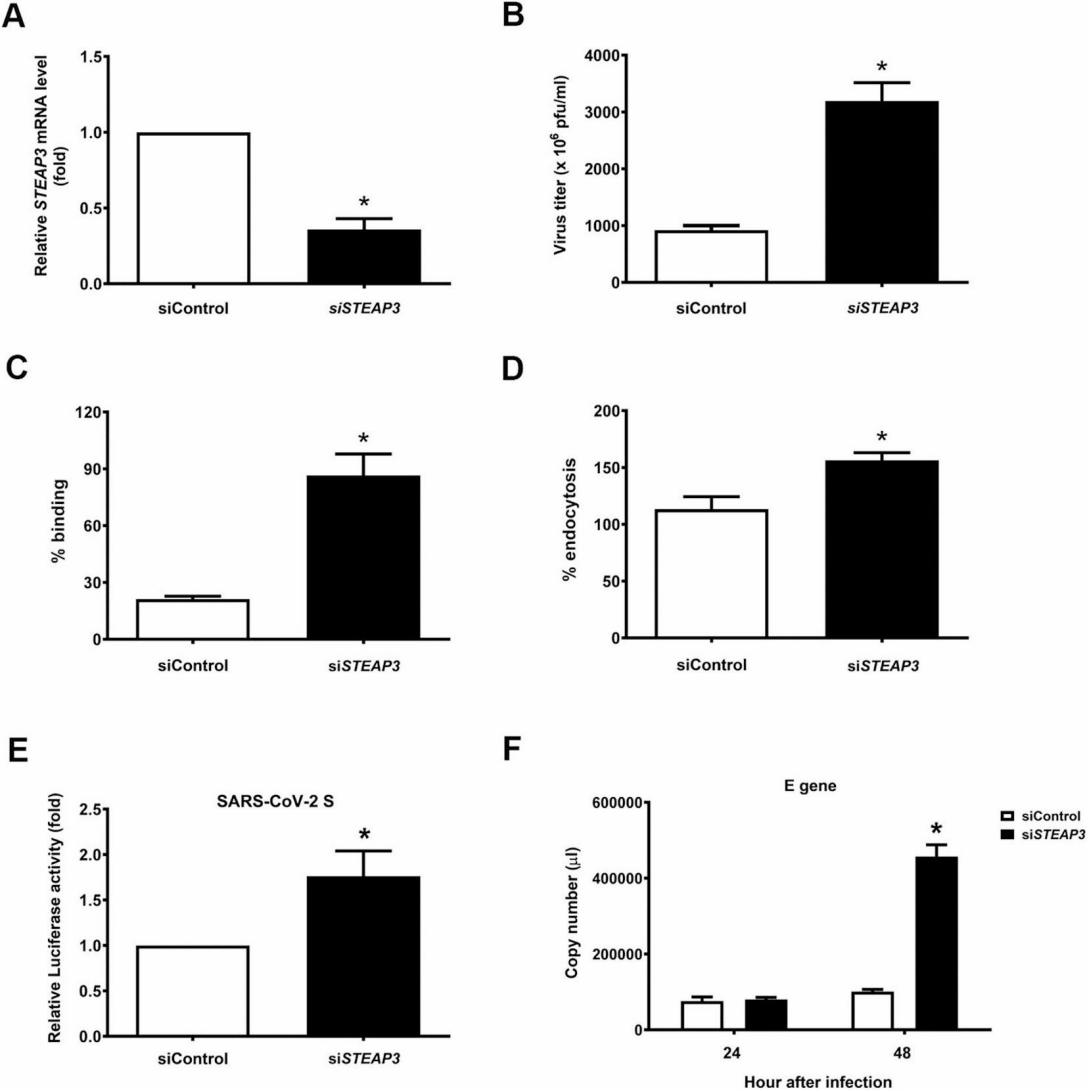
*STEAP3* knockdown enhances virus infection by modulating viral entry. (**A**) The *STEAP3* mRNA level was measured in RD cells transfected with control or *STEAP3*-targeting siRNA. (**B**) The virus titer was measured from siRNA-transfected RD cells infected with EV-A71 (MOI = 1) for 48 h. (**C**) siControl or si*STEAP3*-transfected RD cells were incubated with EV-A71 (MOI = 1) at 4 °C for 1 h. The total viral RNA was collected and measured using a real-time polymerase chain reaction (PCR). (**D**) siControl or si*STEAP3*-transfected RD cells were infected with EV-A71 (MOI = 1) at 4 °C for 1 h and at 37 °C for another 1 h. The total viral RNA was collected and analyzed using real-time PCR. (**E**) Caco-2 cells were transfected with control or *STEAP3*-targeting siRNA and infected with SARS-CoV-2 S entry virus (1.1 × 10⁶ relative infection units) for 24 h. The total cell lysate was collected, and the luciferase activity was measured. Luciferase activity was quantified by the luciferase activity/protein concentration ratio (*n* = 3). (**F**) siControl or si*STEAP3*-transfected calu-3 cells were infected with SARS-CoV-2 (MOI = 10^−1^) for the indicated times. The viral RNA was extracted and analyzed using real-time PCR (*n* = 3). *p < 0.05 versus siControl-transfected cells using the student’s *t*-test. All data are shown as the mean (± s.d.).

STEAP3 functions as a ferrireductase, facilitating the reduction of Fe³⁺ to ferrous iron (Fe²⁺), thereby playing a pivotal role in iron homeostasis. Notably, *STEAP3*-deficient mice exhibit severe hypochromic microcytic anemia and abnormal iron distribution in both tissues and serum^3^. FAC, consisting of ferric iron and citrate, has been reported to suppress infections caused by (EV-A71, influenza A virus, human immunodeficiency virus (HIV), and Zika virus (ZIKV)^18^. However, the precise molecular mechanisms underlying FAC’s antiviral activity remain unclear. Given STEAP3’s critical role in iron metabolism, we investigated its involvement in FAC-mediated antiviral effects. As illustrated in Fig. 2A, downregulation of *STEAP3* in RD cells reversed FAC’s suppressive effect on virus-induced cytopathic effects (CPE). Additionally, FAC treatment led to a reduction in viral capsid protein levels in EV-A71-infected cells. However, *STEAP3* depletion resulted in an increased expression of viral capsid proteins following FAC treatment (Fig. 2B). Similarly, *STEAP3* knockdown attenuated the inhibitory effect of FAC on viral titers in RD cells at both 12 and 24 h post-infection (Fig. 2C). Since late 2019, SARS-CoV-2 has caused a global pandemic, leading to over 7 million confirmed deaths from COVID-19^9^. Although SARS-CoV-2 primarily infects the lung epithelium, increasing evidence suggests that the gastrointestinal tract may serve as a significant site of infection, as viral RNA is detected more persistently in fecal samples than in respiratory specimen^11^. Therefore, we explored the role of STEAP3 in SARS-CoV-2 infectivity in intestinal epithelial cells.

To assess this, we utilized a SARS-CoV-2 Spike pseudotyped lentivirus (SARS-CoV-2 S entry virus), in which the spike protein serves as the envelope glycoprotein, replacing the commonly used vesicular stomatitis virus glycoprotein (VSV-G). These pseudotyped lentiviruses incorporate a luciferase reporter gene driven by a separate promoter and a GFP tag at the C-terminus of the spike protein, allowing for quantification of viral entry by measuring luciferase activity and GFP fluorescence. As shown in Fig. 1D, FAC treatment reduced luciferase activity in Caco-2 cells, a human colon epithelial cell line, whereas *STEAP3* knockdown diminished this inhibitory effect (Supplementary Figs. S1 and S20). Given that FAC suppresses viral infection, these findings indicate that STEAP3 plays a key role in modulating FAC-induced antiviral activity.

### STEAP3 suppressed viral infection by regulating viral entry

**Fig. 3:**
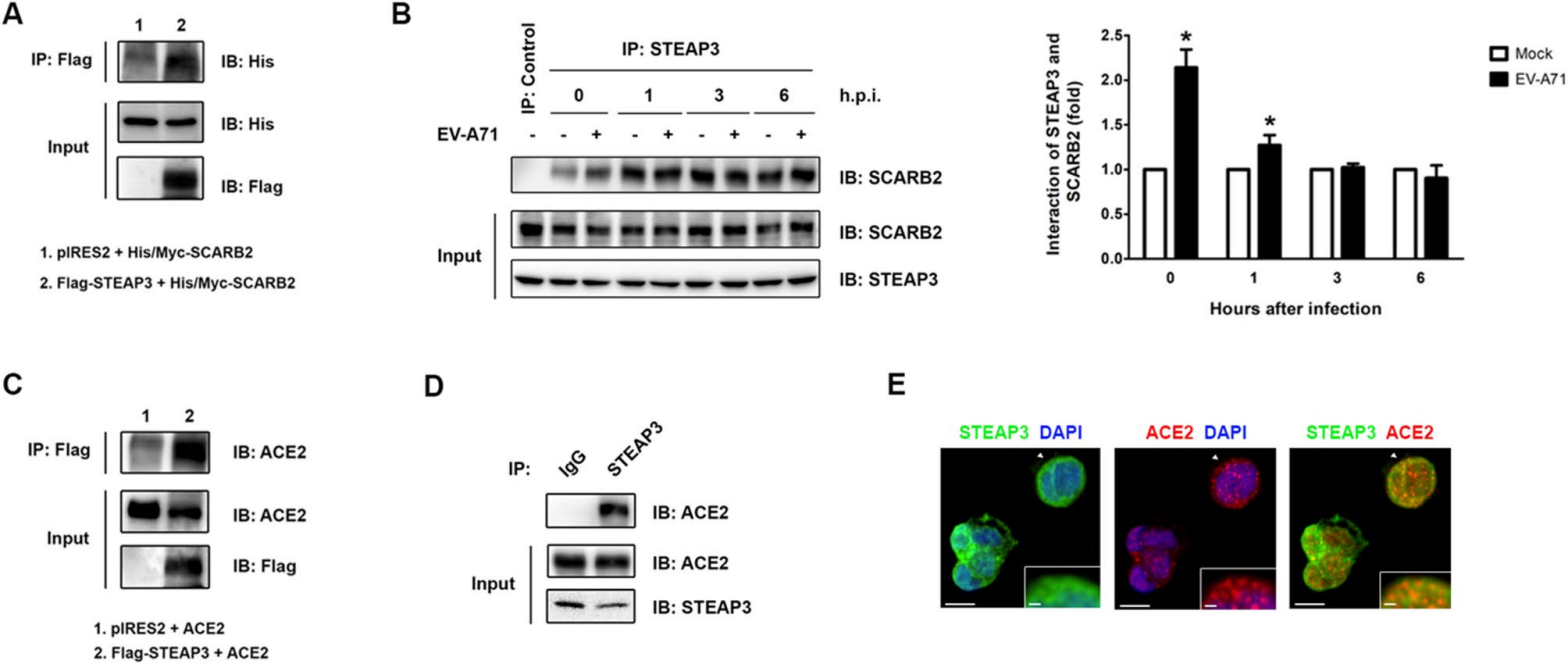
STEAP3 interacts with the viral receptor during viral entry. (**A**) HEK293T cells were transfected with pIRES2-Flag-*STEAP3* and pcDNA3.1-His/Myc-*SCARB2* for 48 h. The total cell lysate was collected, and an IP assay was performed using anti-Flag antibody and separated by SDS-PAGE. The interaction of His/Myc-*SCARB2* was analyzed using anti-His antibody. (**B**) The total cell lysate was collected from RD cells infected with EV-A71 (MOI = 10^−3^) for various intervals and subjected to IP assay with anti-STEAP3 or anti-IgG antibodies. The immunoprecipitates were then separated and analyzed with anti-SCARB2 antibody. The interaction between STEAP3 and SCARB2 was quantified by the ratio of SCARB2 expression in the precipitates to the expression of SCARB2 in total cell lysates (*n* = 3). *p < 0.05 compared to RD cells without EV-A71 infection. Results are presented as the mean percentage (± s.d.). (**C**) ACE2-HEK293T cells were transfected with pIRES2 empty vector or pIRES2-Flag-*STEAP3* for 48 h. The total cell lysate was collected and subjected to an IP using anti-Flag antibody. The binding of ACE2 and STEAP3 was further analyzed in the precipitates by western blot using anti-ACE2 antibody. (**D**) The total cell protein was extracted from Caco-2 cells and immunoprecipitated with anti-STEAP3 or control IgG antibodies. The interactions of ACE2 in anti-STEAP3 precipitates were further analyzed using anti-ACE2 antibody. (**E**) Colocalization between ACE2 and STEAP3 in Caco-2 cells was detected by double immunofluorescence staining with anti-ACE2 (red) and anti-STEAP3 (green) antibodies and counterstained with DAPI (blue). Images were visualized using an Andor Dragonfly confocal microscope. Scale bar = 10 μm. The highlighted area in the inset represents a 2.5-fold magnification of the region indicated by the arrowhead. Scale bar = 1.6 μm. The uncropped and unprocessed blots are presented in Supplementary Fig. S17.

Since STEAP3 regulates iron homeostasis under physiological conditions and FAC induces iron overload, leading to reactive oxygen species-mediated apoptosis^19,20^, we sought to investigate the role of STEAP3 in viral infection in the absence of FAC supplementation. Consequently, decreased *STEAP3* expression significantly increased the viral titer in RD cells after EV-A71 infection (Fig. 3A, B). As STEAP3 possesses a six-transmembrane domain at its C-terminus and is predominantly localized to the plasma membrane, it may play a role in regulating viral infection at an early stage-viral entry. To determine whether STEAP3 participates in viral entry, which involves virus-receptor binding and receptor-mediated endocytosis, we performed virus binding and internalization assays. In the virus-binding assay, RD cells were incubated with the EV-A71 at 4 °C to allow virus-receptor binding and prevent receptor-mediated internalization. The binding of the virus to RD cells was determined by detecting viral RNA in the cells. As shown in Fig. 3B, EV-A71 virus binding dramatically increased in *STEAP3*-downregulated cells compared to that in control cells. In addition, the EV-A71 and RD cells were incubated at 4 °C and then at 37 °C to facilitate viral internalization. EV-A71 viral internalization was slightly increased in RD cells transfected with *STEAP3-*targeting siRNA. These findings were further validated by reconstituting STEAP3 in *STEAP3*-deficient cells (Supplementary Fig. S2), where overexpression of *STEAP3* reversed the enhanced virus binding and internalization.

To further confirm the influence of STEAP3 at the early stage of viral infection, we utilized the SARS-CoV-2 S entry virus to investigate its effects on SARS-CoV-2 entry. As shown in Fig. 3E, downregulation of *STEAP3* in Caco-2 cells significantly increased luciferase activity (Fig. 3E; Supplementary Figs. S3 and S21). To further investigate the effect of STEAP3 on SARS-CoV-2 infection, *STEAP3*-downregulated or control calu-3 cells were infected with live SARS-CoV-2 virus (Supplementary Fig. S4). After SARS-CoV-2 infection for 48 h, the viral RNA levels increased considerably in calu-3 cells (Fig. 3F). Taken together, these findings indicate that STEAP3 suppresses viral infection, potentially by impeding viral entry.

### Enhanced binding of STEAP3 to viral receptors at the early stage of viral infection

**Fig. 4:**
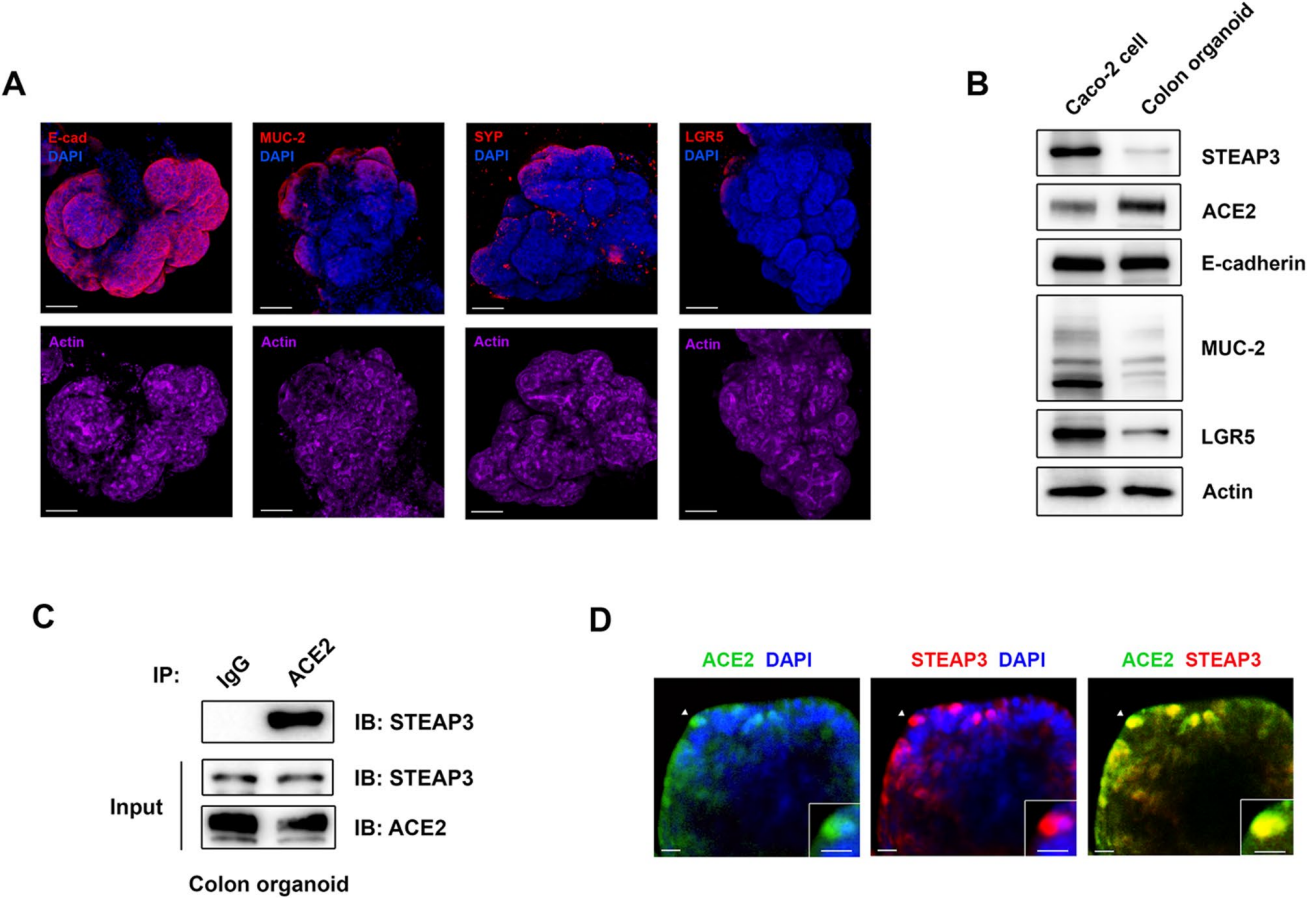
Human colon organoids revealed the binding of STEAP3 to the viral receptor. (**A**) Human colon organoids were derived from tumor specimens of patients with colon cancer. The expression of specific markers of human intestinal epithelial cells, including enterocytes (E-cadherin [E-cad]), goblet cells (mucin [MUC-2]), enteroendocrine cells (synaptophysin [SYP]), and stem cells (leucine-rich repeat-containing G-protein-coupled receptor 5 [LGR5]), was detected and visualized in these organoids using immunofluorescence staining with the indicated antibodies, followed by confocal microscopy and three-dimensional image reconstruction. Scale bar = 100 μm. (**B**) The expression levels of STEAP3, the viral receptor ACE2, and specific markers of human intestinal epithelial cells in Caco-2 cells and human colon organoids were analyzed using immunoblotting with the indicated antibodies. (**C**) The total cell lysate was extracted from human colon organoids and subjected to IP assay with anti-ACE2 or anti-IgG antibodies. The binding of ACE2 to STEAP3 was further analyzed using anti-STEAP3 antibody. (**D**) Colocalization between ACE2 and STEAP3 in colon organoids was detected by double immunofluorescence staining with anti-ACE2 (green) and anti-STEAP3 (red) antibodies and visualized using an Andor Dragonfly confocal microscope. Scale bar = 25 μm. The highlighted area in the inset (arrow) was magnified two-fold. Scale bar = 20 μm. The uncropped and unprocessed immunoblot images are presented in Supplementary Fig. S18.

Previously, we demonstrated that STEAP3 modulated viral infection at the entry stage (Fig. 3). Given that STEAP3 is primarily localized to the plasma membrane and has been shown to interact with epidermal growth factor receptor (EGFR), modulating its nuclear translocation to activate gene expression associated with cancer cell proliferation^4^, it is plausible that STEAP3 may similarly interact with viral receptors to influence viral binding. Therefore, to validate the interaction between STEAP3 and SCARB2, a receptor for EV-A71 binding, Flag-STEAP3 and His/Myc-SCRAB2 were ectopically expressed in HEK293T cells. After IP assay with an anti-Flag antibody to purify the Flag-STEAP3 precipitates, the His/Myc-SCARB2 signal was detected by western blot analysis with an anti-His antibody (Fig. 4A). To further determine whether STEAP3 physically interacts with SCARB2 in cells, total cell lysates were collected from RD cells infected with the EV-A71 for various durations, followed by an IP assay. The interaction between STEAP3 and SCARB2 increased at 0 and 1 h post-EV-A71 infection. Furthermore, this interaction gradually decreased over time, suggesting that STEAP3 binds to viral receptors during the early stage of viral infection (Fig. 4B). Since SARS-CoV-2 primarily utilizes the angiotensin-converting enzyme 2 (ACE2) receptor for cellular entry, the interaction between STEAP3 and ACE2 was also evaluated. As shown in Fig. 4C, binding between Flag-STEAP3 and ACE2 was observed in ACE2-HEK293T cells following IP with an anti-Flag antibody and immunoblotting with an anti-ACE2 antibody. Moreover, their physical interaction was also detected in Caco-2 cells (Fig. 4D). Immunofluorescence staining of STEAP3 and ACE2 in Caco-2 cells further confirmed their colocalization. These findings provide strong evidence that STEAP3 interacts with viral receptors, with this interaction significantly increasing during the early stage of viral infection. Therefore, it is reasonable to suggest that STEAP3 inhibits viral infection by modulating its interaction with viral receptors.

### Colon organoids support the interaction between STEAP3 and the viral receptor

To accurately investigate the role of STEAP3 in viral infection within human intestinal tissues, we generated colon organoids derived from specimens of colon cancer patients. Distinct cell types present in colon tissues were characterized in colon organoids by detecting specific markers of intestinal epithelial cells, including E-cadherin (E-cad) for enterocytes, mucin-2 (MUC2) for goblet cells, synaptophysin (SYP) for enteroendocrine cells, and leucine-rich repeat-containing G-protein-coupled receptor 5 (LGR5) for stem cells. As shown in Fig. 4A and Supplementary Figs. S5–S8, colon organoids exhibited an intact ultrastructural organization, and distinct intestinal epithelial cell types were differentially represented in colon organoids. Compared to Caco-2 cells, a commonly used human intestinal epithelial cell line, colon organoids exhibited lower expression of intestinal markers and STEAP3. However, the expression of the viral receptor ACE2 was elevated in colon organoids. These differential expression patterns raise concerns about the fidelity of using human epithelial cell lines as models for investigating the molecular mechanisms underlying viral infection or for screening the efficacy of inhibitors and neutralizing antibodies. The interaction between STEAP3 and the viral receptor ACE2 was confirmed in colon organoids, along with their colocalization (Fig. 4C, D).

### A 3D-printed 27-well chip with colon organoids effectively evaluated the impact of STEAP3 on viral infectivity

**Fig. 5:**
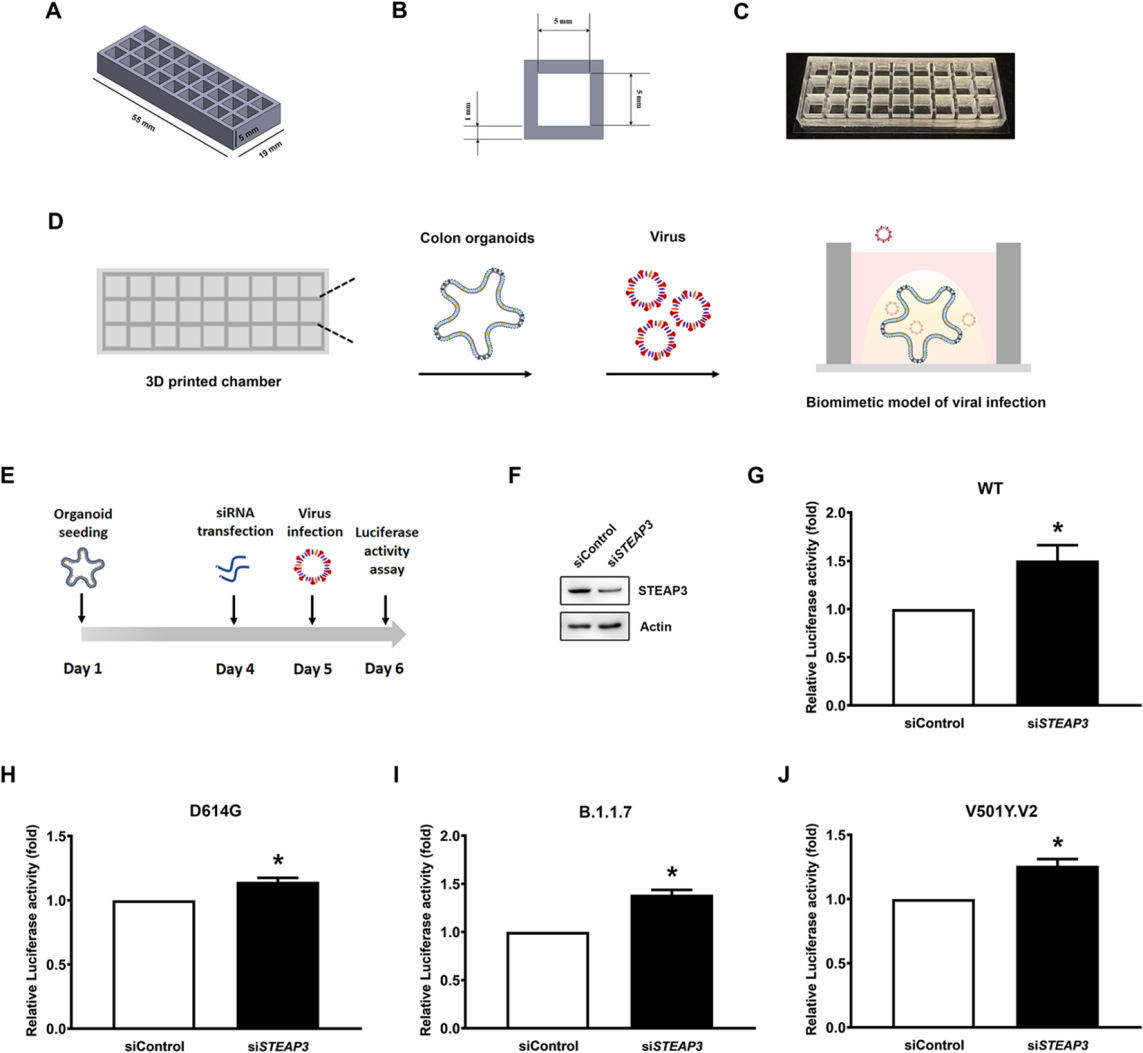
Colon organoids-on-chips for high-throughput screening of STEAP3’s inhibitory effects on viral infectivity. (**A**) Schematic showing the dimensions of the 3D-printed chip. (**B**) Top view of an individual well in the chip. (**C**) A graphic illustrating the actual chip. (**D**) Experimental procedures for constructing a biomimetic model of virus-infected colon organoids in the chip. (**E**) Detailed protocol for evaluating the effects of STEAP3 on viral infection in colon organoids-on-chip. Colon organoids were seeded in the chip and transfected with siControl or si*STEAP3* siRNA for 24 h. The siRNA-transfected organoids were then infected with the SARS-CoV-2 S entry virus for another 24 h. Total protein lysates were extracted from the organoids and subjected to the luciferase activity assay. (**F**) The expressional levels of STEAP3 in control or *STEAP3* knockdown colon organoids. The uncropped and unprocessed blots are provided in Supplementary Fig. S19. (**G-J**) Colon cancer organoids were infected with a SARS-CoV-2 S entry virus encoding a spike protein in its wild-type form or with mutations at amino acid 614 (G614D), eight mutations (B.1.1.7), or two deletions (V501Y.V2) for 24 h. Luciferase activity was measured and quantified as the ratio of luciferase activity to protein concentration (*n* = 3). *p < 0.05 versus siControl-transfected cells using the student’s *t*-test. All data are shown as the mean (± s.d.).

To overcome the limitations of conducting parallel experiments with organoids embedded in Geltrex gel, we fabricated a 27-well chip using MiiCraft BV007a Clear resin an elastomeric polymer known for its biocompatibility, chemical stability, and biodegradation resistance widely used in biomedical applications^21^. The chip was 3D-printed using a MiiCraft Ultra printer and mounted onto a 60 mm × 24 mm cover glass slide with a thickness of 1 mm. As depicted in Fig. 5A, B, the chip has dimensions of 55 mm (L) × 19 mm (W) × 5 mm (H) and contains 27 wells, each measuring 5 mm (L) × 5 mm (W) × 5 mm (H). This chip allows for 27 individual experiments or 9 experiments performed in triplicate within the same batch, thereby reducing variability among different experimental batches (Fig. 5C).

To model virus-infected human intestinal tissue, human colon organoids were derived from tissue specimens of colon cancer patients and cultured in Geltrex gel with intestinal differentiation medium. Once the colon organoids differentiated, forming an ultrastructural intestinal organization and distinct intestinal cell types, they were subsequently infected with the virus (Fig. 5D). To examine the role of STEAP3 in viral infection using the colon organoids-on-chip model, STEAP3 was first silenced in colon organoids using si*STEAP3* siRNA transfection, followed by transduction with SARS-CoV-2 S entry viruses. Luciferase activity was measured in protein lysates extracted from the colon organoids, and the ratio of luciferase activity to total protein content was used to assess viral infectivity. As shown in Fig. 5F, G, knockdown of *STEAP3* obviously increased the infection of wild-type (WT) SARS-CoV-2 S entry viruses in colon organoids. However, mutations occur continuously in SARS-CoV-2, resulting in the emergence of new variants that increase its transmissibility and ability to escape immune responses; specifically, mutations at the SARS-CoV-2 spike protein could diminish the efficacy of the vaccine and increase the risk of reinfection. The D614G variant was established from the G614 variant, in which aspartate at position 23,402 was substituted with glycine. Compared with the G614 variant, the G614D variant has high infectivity and spread rapidly during early 2020^22^. The B.1.1.7 (20I/501Y. V1) variant, consisting of eight mutations in the spike protein, has a high binding affinity for the ACE2 receptor, resulting in a high transmission rate in the UK^23^. Subsequently, the V501Y.V2 variant emerged in South Africa, which increased the transmission rate by approximately 50% and decreased the efficacy of spike protein-based vaccines^24^. Therefore, we examined whether STEAP3 also modulates the viral infection of SARS-CoV-2 variants. As shown in Figs. 5H, I and J, colon cancer organoids downregulated by si*STEAP3* were vulnerable to infection with the G614D, B.1.1.7, and V501Y.V2 SARS-CoV-2 variants. Based on these findings, we demonstrated the importance of STEAP3 in the infectivity of SARS-CoV-2 and its variants using a 3D model of colon organoid-on-chip model.

### Knockdown of *STEAP3* predominantly enhanced viral infection in enterocytes and enteroendocrine cells of the intestinal epithelium

**Fig. 6:**
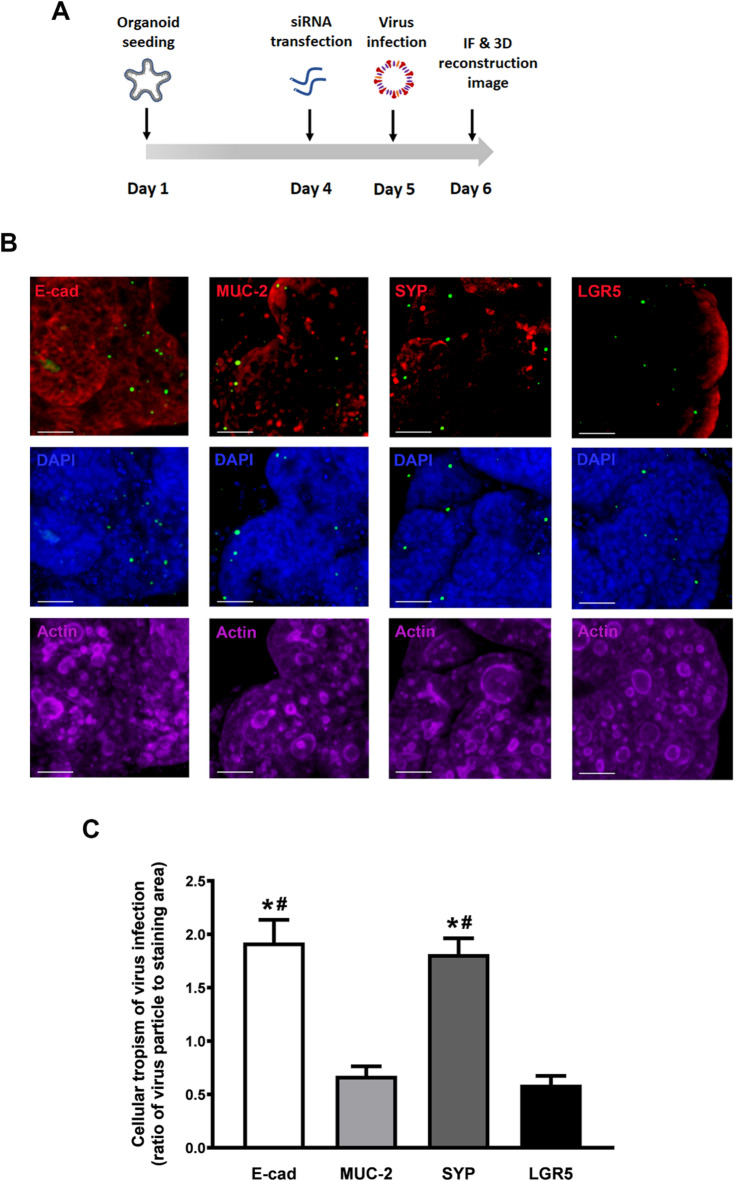
*STEAP3*-deficient colon organoids reveal the cellular tropism of the intestinal epithelium to viral infection. (**A**) Protocol for studying the knockdown of *STEAP3* on cellular tropism of viral infection in colon organoids-on-chip. Colon organoids cultivated in the chip were transfected with siControl or si*STEAP3* siRNA for 24 h and then infected with the SARS-CoV-2 S entry virus for another 24 h. The colon organoids-on-chip were then fixed and immunofluorescence-stained for intestinal cell markers. The images were visualized and 3D-reconstructed using confocal microscopy with 2 μm *z*-stack sections. (**B**) 3D images displayed the GFP signals (green) from the SARS-CoV-2 S entry virus carrying a GFP tag in colon organoids stained with distinct intestinal markers (red) of E-cad, mucin-2 (MUC-2), SYP, and LGR5. Scale bar = 50 μm. (**C**) The cellular tropism of viral infection was detected and measured according to the ratio of virus particles to staining area of individual markers of intestinal epithelium. The virus particles were determined by calculating the dot signal of GFP. The five different fields of images were measured and quantified by one-way ANOVA analysis. *p < 0.05, versus MUC-2-staining group. ^#^*p* < 0.05, compared to LGR5-staining group.

Human intestinal tissues consist of multiple cell types, each of which may exhibit varying susceptibility to *STEAP3* knockdown in promoting viral infection. We have designed SARS-CoV-2 S entry virus carries a GFP tag at the C-terminus of the spike protein along with luciferase reporter (GFP-SARS-CoV-2 S), allowing its infectivity to be observed and quantified through GFP signal detection. Furthermore, the colon organoids-on-chip were mounted on a 1 mm-thick coverslip to facilitate high-resolution visualization under microscopy. As shown in Fig. 6A, virus-infected colon organoids underwent immunofluorescence staining with specific intestinal epithelial cell markers to identify cell types and were visualized under confocal microscopy with z-stacks to determine their susceptibility to *STEAP3* knockdown in enhancing viral infection. The 3D images reconstructed from *z*-stack data (Fig. 6B) and 3D movies with rotated orthogonal views (Supplementary Figs. S9–S12) demonstrated SARS-CoV-2 S entry viruse infection in specific cell types within colonic organoids. To assess which cell type was more susceptible to viral infection following *STEAP3* deficiency, the ratio of GFP signals from SARS-CoV-2 S entry viruses to the positive staining area of individual intestinal markers was calculated. In Fig. 6C, the presence of more GFP-virus particles in E-cadherin (E-cad) and synaptophysin (SYP) staining areas indicates that enterocytes and enteroendocrine cells were more prone to viral infection after *STEAP3* knockdown.

### Vascularized colon organoid-on-chip model revealed that *STEAP3* downregulation enhanced viral infection within the vascular circulation

**Fig. 7:**
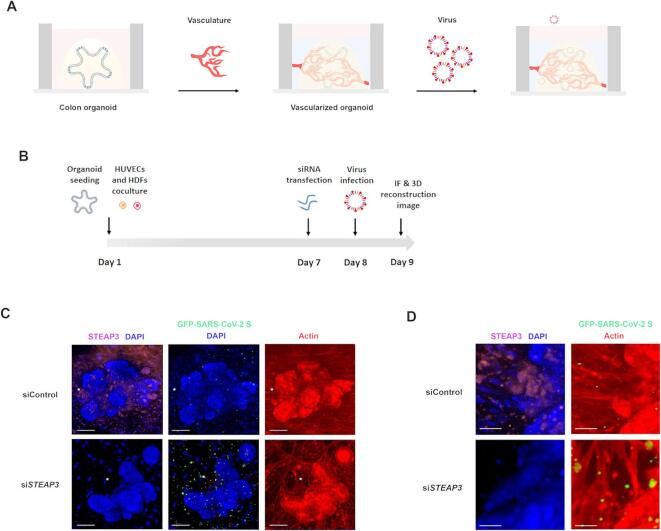
Knockdown of *STEAP3* enhanced viral circulation in the vascularized colon organoid. (**A**) Experimental procedures for constructing a biomimetic chip of virus-infected vascularized colon organoids. (**B**) Detailed protocol for detecting the enhancement of *STEAP3*-deficient on viral infection in vascularized organoids-on-chip. After colon organoids were seeded in the chip, HUVECs and HDFs were subsequently cultured in the matrix of fibrin and AdECM and applied onto organoids. After cultivation for 7 days, the vascular network was formed, vascularized organoids were transfected with siRNA targeting to control or *STEAP3* for 24 h and then infected with SARS-CoV-2 S entry virus for an additional 24 h. The viral infectivity was visualized by using confocal microscopy. (**C**) Vascularized organoids were fixed and immunofluorescence-stained for STEAP3 (purple) and actin (red). Nuclei (blue) were counterstained with DAPI. 3D images were reconstructed using confocal microscopy with 2 μm *z*-stack sections. Scale bar = 50 μm. The asterisk (*) indicates the enlarged region. (**D**) Enlarged images of the region marked by the asterisk (*) in (c). Scale bar = 25 μm.

The interaction between cells and their surrounding microenvironment is crucial for maintaining physiological homeostasis in normal tissues and promoting tumor growth, while also regulating cell behavior and phenotypes^25^. Among the various components of the microenvironment, the vasculature is the most prevalent, covering a surface area of 4,000–7,000 m² and extending throughout all organs. Otherwise, a hallmark of viral infection in endothelial cells is the dissemination of virus particles through circulation, leading to the infection of multiple organs^26^. To accurately model human organs, we developed a colon organoid-on-chip system with integrated vasculature. As illustrated in Fig. 7A, B, colon organoids were co-cultured with HUVECs and HDFs to generate vascularized organoids. These vascularized organoids were subjected to *STEAP3* knockdown and subsequently infected with GFP-tagged SARS-CoV-2 S entry virus. Notably, *STEAP3* knockdown significantly enhanced viral infection, as evidenced by an increased GFP signal in *STEAP3*-deficient vascularized organoids, particularly in regions adjacent to the organoids (Fig. 7C). An enlarged view of vascular networks, marked by stars in Fig. 7C, D, revealed a higher presence of GFP-tagged SARS-CoV-2 S entry virus within vascular lumens in *STEAP3*-deficient organoids compared to control organoids. This observation suggests that STEAP3 deficiency may promote viral infection via vascular circulation. Collectively, these findings suggests that *STEAP3* knockdown increases the susceptibility of both tissues and their surrounding microenvironment to viral infection.

## Discussion

Our findings suggest that *STEAP3* deficiency facilitates viral entry, leading to increased viral infection in human cells, as demonstrated in Fig. 2. This provides strong evidence for the regulatory role of STEAP3 in viral entry, which has not been previously reported. A prior study revealed that STEAP3 influences the replication of porcine reproductive and respiratory syndrome virus (PRRSV), a pathogen that causes porcine reproductive and respiratory syndrome and affects swine health. In that study, PRRSV infection of MARC-145 cells, a monkey embryonic kidney epithelial cell line, resulted in decreased STEAP3 expression^27^, which is not shown in our results. These differences in STEAP3 regulation suggest that STEAP3 may exhibit species-specific functions and distinct roles in response to different viral infections.

Although FAC (100 µM) treatment for 24 h has been reported to inhibit influenza A virus infection in A549 cells^18^, it also impairs cell proliferation and disrupts intracellular iron homeostasis, resulting in oxidative stress injury^28^. In our study, we further demonstrated that FAC possesses antiviral activity against SARS-CoV-2 (Fig. 1D), in addition to its previously reported effects against other viruses^18^. Nevertheless, the downregulation of *STEAP3* attenuated the antiviral effect of FAC on viral infection (Fig. 1), suggesting that STEAP3 plays a role in FAC-mediated antiviral activity. However, our data showed that *STEAP3* knockdown significantly enhanced viral infection in the absence of FAC supplementation, as demonstrated in Fig. 2, thereby mitigating the cytotoxic effects of FAC on cells.

STEAP3, primarily localized at the plasma membrane and involved in maintaining iron homeostasis, has been reported to interact with the EGFR to regulate STAT3-mediated cell proliferation^4^. Given that the recognition and interaction of plasma membrane-localized viral receptors with the virus is a critical step in the initial phase of infection^29^, we found that STEAP3 physically interacts with viral receptors, with their binding increasing during the early stage of viral infection (Fig. 3). These suggest that STEAP3 may inhibit viral entry by interacting with viral receptors.

The conventional 2D model fails to accurately represent the physiological behaviors of organs or tumors within the human body, including cell-cell interactions and communication between cells and their surrounding microenvironment^13,14^. This limitation reduces the reliability of experimental results and their translational relevance to clinical trials. 3D model of organoid that faithfully recapitulates key characteristics of tissue in human body has become a promising tool in studying disease progression and the interplay between microorganisms and host^15^. Emerging evidence has shown that patients with cancer, including those with colon cancer, have a higher susceptibility to COVID-19^30^. Notably, ACE2 is highly expressed in colon cancer tissues compared to normal colon tissues. This elevated ACE2 expression may predispose tumor tissues to increased viral entry. Additionally, colon cancer patients with COVID-19 have been reported to exhibit more severe symptoms, such as lymphopenia, elevated respiratory rates, and increased hypersensitive C-reactive protein levels, compared to COVID-19 patients without cancer^31,32^. These findings underscore the heightened vulnerability of this patient population and emphasize the need for targeted investigations into the interactions between SARS-CoV-2 and cancerous tissues. In our study, we utilized tumor organoids to more accurately investigate viral susceptibility, host responses, and potential therapeutic interventions in this high-risk group.

We have developed a colon organoid-on-a-chip model using organoids derived from tissue specimens of patients with colon cancer to investigate the role of STEAP3 in viral infection within intestinal tissue. The intestinal epithelium is composed of distinct cell types, including enterocytes, goblet cells, enteroendocrine cells, and stem cells. The expression of intestinal epithelial cell markers differs between colon epithelial cell lines and colon epithelial organoids, with notable variations in the expression levels of STEAP3 and the viral receptor ACE2 (Fig. 4B), which may result different underlying mechanisms in response to viral infection. The expression patterns of MUC2 and LGR5 differ between the colon cancer cell line (Caco-2 cells) and colon organoids (Fig. 4B and Supplementary Figs. S13 and S22), highlighting the limitations of using 2D cultures as a model for studying viral infection.

To enable organoid cultivation within a 3D culture system and support high-throughput screening of multiple experimental conditions on a single platform, we designed and fabricated a custom chip comprising 27 wells mounted on a 1 mm-thick glass coverslip (Figs. 5A, B, C, D). Commercially available chamber slides typically contain fewer wells (e.g., 12-well formats) or, in the case of 96-well plates, are not well-suited for 3D organoid culture and high-resolution microscopic imaging. Given that the SARS-CoV-2 S entry viruses used in this study were engineered to express dual reporters—luciferase and GFP—the application of these viruses to the 27-well organoid chip enabled both real-time visualization of viral infectivity via fluorescence microscopy and quantitative assessment through luciferase assays. This integrated platform substantially improves the accuracy and throughput of investigating viral infection mechanisms and facilitates the screening of antiviral inhibitors or neutralizing antibodies.

As shown in Fig. 5, applying si*STEAP3* siRNA to organoids significantly reduced STEAP3 protein levels and enhanced the luciferase signal from SARS-CoV-2 S entry viruses, indicating that *STEAP3* deficiency promotes viral infection. Additionally, 3D reconstructed images (Fig. 6) showed that enterocytes and enteroendocrine cells of the intestinal epithelium exhibited increased susceptibility to viral infection following *STEAP3* knockdown, as determined by the ratio of viral quantity to the positively stained area of intestinal markers. Consistently, previous studies have identified enterocytes as the primary targets of SARS-CoV-2 infection^33^, which is expected given that enterocytes constitute more than half of intestinal epithelial cells^34^. Notably, our study is the first to demonstrate that cellular tropism for viral infection can be observed following the modulation of specific protein expression levels. Since enterocytes and enteroendocrine cells form the outermost layer of the intestinal epithelium and serve as the first line of defense against viral exposure^12^, the regulation of viral infection by STEAP3 in these cells suggests its potential role as a gatekeeper in antiviral defense.

The interaction between cells and their surrounding microenvironment is essential for preserving the physiological homeostasis of normal tissues and promoting tumor growth. The vasculature is a fundamental component of this microenvironment, playing a critical role in delivering nutrients and oxygen to sustain cellular homeostasis across various organs and tumors^35^. In our previous study, we developed a matrix composed of AdECM and fibrin to facilitate and support the formation of a vascular network^36^. Building upon this approach, we applied this vascular system to colon organoids to construct a vascularized organoid model, enabling the investigation of the effects of *STEAP3* deficiency on viral infection within the vasculature. Following *STEAP3* knockdown, its expression was significantly reduced in vascularized organoids, as shown in Fig. 7C. The knockdown efficiency of STEAP3-targeting siRNA was further validated in colon organoids and HUVECs (Fig. 5F and Supplementary Figs. S14 and S23, respectively). As shown in Fig. 7 and Supplementary Fig. S15, *STEAP3* downregulation led to an increase in viral infection within the vascular network, as evidenced by the enhanced GFP signal within vascular lumens. Collectively, our biomimetic model of vascularized colon organoids provides a precise platform for studying the role of STEAP3 in viral infection within the intestinal epithelium and its surrounding microenvironment. This model enhances our understanding of the complex interplay among viruses, host tissues, and their microenvironment.

## Conclusion

**Fig. 8:**
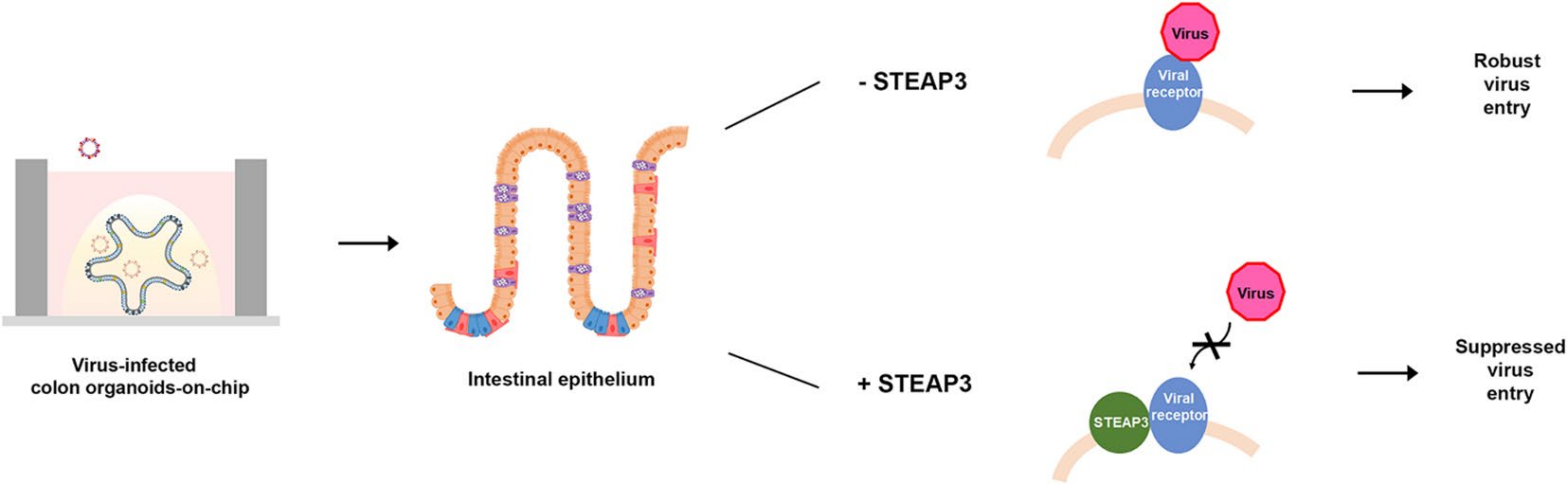
A schematic illustrating the construction of a biomimetic chip of human colon organoids for dissecting the molecular mechanisms by which STEAP3 regulates viral infection in intestinal epithelial cells.

To comprehensively investigate the role of STEAP3 in viral infection, we employed both 3D organoid systems and 2D cell cultures, and established a biomimetic 27-well colon organoid-on-chip platform as an efficient experimental model. By integrating 3D and 2D model systems, we demonstrated that STEAP3 plays an inhibitory role during the viral entry phase, potentially through interactions with viral receptors. As illustrated in Fig. 8, the 3D organoid-on-chip model showed that *STEAP3* knockdown significantly increased viral infectivity, particularly in enterocytes and enteroendocrine cells, while vascularized organoids further revealed enhanced viral dissemination within the vascular lumen. In the 2D cell culture system, we identified the underlying molecular mechanisms, demonstrating that STEAP3 primarily interacts with viral receptors during the early stage of infection, suggesting its antiviral function is mediated through modulation of receptor interactions. Collectively, these findings emphasize the crucial role of STEAP3 in intestinal antiviral defense and highlight the organoid-on-chip platform as a valuable tool for studying host–virus interactions. Beyond viral infections, this platform may also serve to investigate the pathogenesis of other diseases, support drug screening, and facilitate the development of targeted therapeutic strategies.

## Supplementary Information

Below is the link to the electronic supplementary material.

Supplementary Material 1

## Data Availability

All data supporting the findings of this study are included in the manuscript and supplementary information files; additional datasets used and/or analyzed during the current study are available from the corresponding author upon reasonable request.

## References

[1] Grunewald, T. G., Bach, H., Cossarizza, A. & Matsumoto, I. The STEAP protein family: versatile oxidoreductases and targets for cancer immunotherapy with overlapping and distinct cellular functions. *Biol. Cell.* **104** (11), 641–657 (2012). 22804687 10.1111/boc.201200027

[2] Ohgami, R. S. et al. Identification of a ferrireductase required for efficient transferrin-dependent iron uptake in erythroid cells. *Nat. Genet.* **37** (11), 1264–1269 (2005). 16227996 10.1038/ng1658 PMC2156108

[3] Ohgami, R. S. et al. nm1054: a spontaneous, recessive, hypochromic, microcytic anemia mutation in the mouse. *Blood* **106** (10), 3625–3631 (2005). 15994289 10.1182/blood-2005-01-0379 PMC1819405

[4] Wang, L. L. et al. STEAP3 promotes cancer cell proliferation by facilitating nuclear trafficking of EGFR to enhance RAC1-ERK-STAT3 signaling in hepatocellular carcinoma. *Cell. Death Dis.* **12** (11), 1052 (2021). 34741044 10.1038/s41419-021-04329-9 PMC8571373

[5] Shi, H., Lei, S., Xiong, L., Du, S. & Shi, Y. Molecular characterization of STEAP3 in lung squamous cell carcinoma: regulating EGFR to affect cell proliferation and ferroptosis. *Arch. Biochem. Biophys.* **751**, 109842 (2024). 38040224 10.1016/j.abb.2023.109842

[6] Lespagnol, A. et al. Exosome secretion, including the DNA damage-induced p53-dependent secretory pathway, is severely compromised in TSAP6/Steap3-null mice. *Cell. Death Differ.* **15** (11), 1723–1733 (2008). 18617898 10.1038/cdd.2008.104

[7] Amzallag, N. et al. TSAP6 facilitates the secretion of translationally controlled tumor protein/histamine-releasing factor via a nonclassical pathway. *J. Biol. Chem.* **279** (44), 46104–46112 (2004). 15319436 10.1074/jbc.M404850200

[8] Passer, B. J. et al. The p53-inducible TSAP6 gene product regulates apoptosis and the cell cycle and interacts with Nix and the Myt1 kinase. *Proc. Natl. Acad. Sci. U S A*. **100** (5), 2284–2289 (2003). 12606722 10.1073/pnas.0530298100 PMC151332

[9] Huang, Y. et al. Clinical characteristics of laboratory confirmed positive cases of SARS-CoV-2 infection in wuhan, china: A retrospective single center analysis. *Travel Med. Infect. Dis.* **36**, 101606 (2020). 32114074 10.1016/j.tmaid.2020.101606 PMC7102650

[10] Arjmand, B., Ghorbani, F., Koushki, M. & Rezai-Tavirani, M. Gastrointestinal symptoms in patients with mild and severe COVID-19: a scoping review and meta-analysis. *Gastroenterol. Hepatol. Bed Bench*. **13** (4), 321–330 (2020). 33244374 PMC7682965

[11] Wu, Y. et al. Prolonged presence of SARS-CoV-2 viral RNA in faecal samples. *Lancet Gastroenterol. Hepatol.* **5** (5), 434–435 (2020). 32199469 10.1016/S2468-1253(20)30083-2 PMC7158584

[12] Peterson, L. W. & Artis, D. Intestinal epithelial cells: regulators of barrier function and immune homeostasis. *Nat. Rev. Immunol.* **14** (3), 141–153 (2014). 24566914 10.1038/nri3608

[13] von der Mark, K., Gauss, V., von der Mark, H. & Muller, P. Relationship between cell shape and type of collagen synthesised as chondrocytes lose their cartilage phenotype in culture. *Nature* **267** (5611), 531–532 (1977). 559947 10.1038/267531a0

[14] Petersen, O. W., Ronnov-Jessen, L., Howlett, A. R. & Bissell, M. J. Interaction with basement membrane serves to rapidly distinguish growth and differentiation pattern of normal and malignant human breast epithelial cells. *Proc. Natl. Acad. Sci. U S A*. **89** (19), 9064–9068 (1992). 1384042 10.1073/pnas.89.19.9064 PMC50065

[15] Liu, L., Yu, L., Li, Z., Li, W. & Huang, W. Patient-derived organoid (PDO) platforms to facilitate clinical decision making. *J. Transl Med.* **19** (1), 40 (2021). 33478472 10.1186/s12967-020-02677-2 PMC7821720

[16] Morgan, R. G. et al. Optimized delivery of SiRNA into 3D tumor spheroid cultures in situ. *Sci. Rep.* **8** (1), 7952 (2018). 29785035 10.1038/s41598-018-26253-3 PMC5962539

[17] Kung, Y. A. et al. Acyl-Coenzyme A synthetase long-chain family member 4 is involved in viral replication organelle formation and facilitates virus replication via ferroptosis. *mBio* **13** (1), e0271721 (2022). 35038927 10.1128/mbio.02717-21 PMC8764547

[18] Wang, H. et al. Antiviral effects of ferric ammonium citrate. *Cell. Discov*. **4**, 14 (2018). 29619244 10.1038/s41421-018-0013-6 PMC5871618

[19] Li, S. W. et al. Iron overload induced by ferric ammonium citrate triggers reactive oxygen species-mediated apoptosis via both extrinsic and intrinsic pathways in human hepatic cells. *Hum. Exp. Toxicol.* **35** (6), 598–607 (2016). 26224043 10.1177/0960327115597312

[20] Ohgami, R. S., Campagna, D. R., McDonald, A. & Fleming, M. D. The Steap proteins are metalloreductases. *Blood* **108** (4), 1388–1394 (2006). 16609065 10.1182/blood-2006-02-003681 PMC1785011

[21] Miranda, I. et al. Properties and applications of PDMS for biomedical engineering: A review. *J Funct. Biomater* **13**(1). 2–22 (2021). 10.3390/jfb13010002 PMC8788510 35076525

[22] Isabel, S. et al. Evolutionary and structural analyses of SARS-CoV-2 D614G Spike protein mutation now documented worldwide. *Sci. Rep.* **10** (1), 14031 (2020). 32820179 10.1038/s41598-020-70827-z PMC7441380

[23] Shen, X. et al. SARS-CoV-2 variant B.1.1.7 is susceptible to neutralizing antibodies elicited by ancestral Spike vaccines. *Cell. Host Microbe*. **29** (4), 529–539 (2021). e523. 33705729 10.1016/j.chom.2021.03.002 PMC7934674

[24] Sabino, E. C. et al. Resurgence of COVID-19 in manaus, brazil, despite high Seroprevalence. *Lancet* **397** (10273), 452–455 (2021). 33515491 10.1016/S0140-6736(21)00183-5 PMC7906746

[25] Nishida-Aoki, N. & Gujral, T. S. Emerging approaches to study cell-cell interactions in tumor microenvironment. *Oncotarget* **10** (7), 785–797 (2019). 30774780 10.18632/oncotarget.26585 PMC6366828

[26] Fosse, J. H., Haraldsen, G., Falk, K. & Edelmann, R. Endothelial cells in emerging viral infections. *Front. Cardiovasc. Med.* **8**, 619690 (2021). 33718448 10.3389/fcvm.2021.619690 PMC7943456

[27] Yuan, C., Guan, K. & Zhang, G. STEAP3 inhibits Porcine reproductive and respiratory syndrome virus replication by regulating fatty acid and lipid droplet synthesis. *Vet Sci* **12**(2). 147–158 (2025). 10.3390/vetsci12020147 PMC11861627 40005907

[28] Wu, W., Geng, Z., Bai, H., Liu, T. & Zhang, B. Ammonium ferric citrate induced ferroptosis in non-small-cell lung carcinoma through the Inhibition of GPX4-GSS/GSR-GGT axis activity. *Int. J. Med. Sci.* **18** (8), 1899–1909 (2021). 33746607 10.7150/ijms.54860 PMC7976582

[29] Maginnis, M. S. Virus-receptor interactions: the key to cellular invasion. *J. Mol. Biol.* **430** (17), 2590–2611 (2018). 29924965 10.1016/j.jmb.2018.06.024 PMC6083867

[30] Niu, P., Lei, F. & Gu, J. Colorectal cancer and COVID-19: Do we need to raise awareness and vigilance? *Cancer* **127**(6), 979–980. (2021). 10.1002/cncr.33217 PMC8014734 33498093

[31] Ren, P., Gong, C. & Ma, S. Evaluation of COVID-19 based on ACE2 expression in normal and cancer patients. *Open. Med. (Wars)*. **15** (1), 613–622 (2020). 33336018 10.1515/med-2020-0208 PMC7712168

[32] Liu, C. et al. High expression of ACE2 and TMPRSS2 and clinical characteristics of COVID-19 in colorectal cancer patients. *NPJ Precis Oncol.* **5** (1), 1 (2021). 33479506 10.1038/s41698-020-00139-y PMC7820314

[33] Zhao, X. et al. Human intestinal organoids recapitulate enteric infections of enterovirus and coronavirus. *Stem Cell. Rep.* **16** (3), 493–504 (2021). 10.1016/j.stemcr.2021.02.009 PMC7940440 33626333

[34] Cheng, H. & Leblond, C. P. Origin, differentiation and renewal of the four main epithelial cell types in the mouse small intestine. I. Columnar cell. *Am. J. Anat.* **141** (4), 461–479 (1974). 4440632 10.1002/aja.1001410403

[35] Butler, J. M., Kobayashi, H. & Rafii, S. Instructive role of the vascular niche in promoting tumour growth and tissue repair by angiocrine factors. *Nat. Rev. Cancer*. **10** (2), 138–146 (2010). 20094048 10.1038/nrc2791 PMC2944775

[36] Shih, Y. Y., Kao, C. W., Jhong, Y. R., Chen, Y. A. & Chen, Y. W. Synergistic effects of fibrin-enriched adipose decellularized extracellular matrix (AdECM) and microfluidic model on vascularization. *RSC Adv.* **14** (46), 34143–34155 (2024). 39469019 10.1039/d4ra05573j PMC11513771

